# A Proteomic- and Bioinformatic-Based Identification of Specific Allergens from Edible Insects: Probes for Future Detection as Food Ingredients

**DOI:** 10.3390/foods10020280

**Published:** 2021-01-30

**Authors:** Annick Barre, Carole Pichereaux, Mathias Simplicien, Odile Burlet-Schiltz, Hervé Benoist, Pierre Rougé

**Affiliations:** 1 UMR 152 PharmaDev, Institut de Recherche et Développement, Faculté de Pharmacie, Université Paul Sabatier, 35 Chemin des Maraîchers, 31062 Toulouse, France; annick.barre@univ-tlse3.fr (A.B.); simplicien.mathias@gmail.com (M.S.); herve.benoist@ird.fr (H.B.); 2 Fédération de Recherche (FR3450), Agrobiosciences, Interactions et Biodiversité (AIB), CNRS, 31326 Toulouse, France; Carole.Pichereaux@ipbs.fr; 3 Institut de Pharmacologie et Biologie Structurale (IPBS), Université de Toulouse UPS, CNRS, 31077 Toulouse, France; Odile.Schiltz@ipbs.fr

**Keywords:** edible insect proteins, insect food allergens, food allergy, cricket, giant milworm, migratory locust, silkworm, yellow mealworm, palm weevil, allergen structure diversity, structure-function relationships, allergen detection

## Abstract

The increasing development of edible insect flours as alternative sources of proteins added to food and feed products for improving their nutritional value, necessitates an accurate evaluation of their possible adverse side-effects, especially for individuals suffering from food allergies. Using a proteomic- and bioinformatic-based approach, the diversity of proteins occurring in currently consumed edible insects such as silkworm (*Bombyx mori*), cricket (*Acheta domesticus*), African migratory locust (*Locusta migratoria*), yellow mealworm (*Tenebrio molitor*), red palm weevil (*Rhynchophorus ferrugineus*), and giant milworm beetle (*Zophobas atratus*), was investigated. Most of them consist of phylogenetically-related protein allergens widely distributed in the different groups of arthropods (mites, insects, crustaceans) and mollusks. However, a few proteins belonging to discrete protein families including the chemosensory protein, hexamerin, and the odorant-binding protein, emerged as proteins highly specific for edible insects. To a lesser extent, other proteins such as apolipophorin III, the larval cuticle protein, and the receptor for activated protein kinase, also exhibited a rather good specificity for edible insects. These proteins, that are apparently missing or much less represented in other groups of arthropods, mollusks and nematods, share well conserved amino acid sequences and very similar three-dimensional structures. Owing to their ability to trigger allergic responses in sensitized people, they should be used as probes for the specific detection of insect proteins as food ingredients in various food products and thus, to assess their food safety, especially for people allergic to edible insects.

## 1. Introduction

The increasing use of insects as food ingredients, developed at the same time that progress has been achieved in the factory farming of edible insects such as yellow mealworm (*Tenebrio molitor*), cricket (*Acheta domesticus*, *Gryllus bimaculatus*), or black soldier fly (*Hermetia illucens*) [1,2,3,4,5,6,7,8,9,10,11,12,13,14,15]. However, besides their relevant nutritional properties, products derived from edible insects may also exhibit some potential chemical safety risks due to the persistence of heavy metals or hazardous organic pollutants in the ready-to-eat insects [16,17,18,19,20,21,22], and microbiological and parasitological risks associated to the possible occurrence in edible insects of bacteria or parasites potentially pathogenic for humans [23,24,25,26]. In addition, edible insects may also exhibit deleterious effects on people suffering from allergic disease towards insects and, more broadly, towards arthropods (acari, crustaceans, insects), mollusks and nematods [27]. Accordingly, facing the potential allergenic risk associated to edible insect consumption, adapted methods of detection need to be developed for probing food products containing insect flours, particularly potential insect protein allergens.

Allergic responses associated to entomophagy, i.e., the consumption of edible insects, has been known for a long time and has been recently reviewed in details [28,29,30,31,32,33]. More recently, an anaphylactic reaction was reported after consumption of yellow mealworm [34], and silkworm pupae [35]. Most of the insect allergens identified as IgE-binding allergens, cross-react with shellfish, mollusk and nematod allergens and thus correspond to IgE-binding cross-reacting pan-allergens, widely distributed in various animal phyla [36,37,38,39,40,41,42,43,44]. Among these cross-reacting allergens, alpha-actin, arginine-kinase (AK), enolase, fructose-1,6-biphosphate aldolase (FPA), glyceraldehyde-3-phopho-deshydrogenase (GAPDH), and tropomyosin, consist of the frequently identified IgE-binding cross-reacting allergens [33]. Other widely distributed proteins such as alpha-amylase, glutathione S-transferase (GST), myosin, paramyosin, triose-phosphate isomerase (TPI), and troponin, also occur as IgE-binding cross-reacting allergens in insects, crustaceans and mollusks [36,39]. Besides pan-allergens, insects might contain much more specific allergens, which could be used as specific probes for the detection of insect flour added either intentionally as a food ingredient or involuntarily as hidden allergens, to food products. Such a detection approach, based on the occurrence of allergens specific for edible insects in food products, could be used as a complementary approach for the genomic detection of insect species/flour/proteins in foods, using e.g., a multiplex polymerase chain reactions (PCR) [45].

The present work was aimed at identifying potential allergens that are more specifically distributed in edible insects, while lacking or weakly represented in other arthropods, mollusks and nematods, to dispose of more specific probes to detect the addition of insect flour to food products.

## 2. Materials and Methods

### 2.1. Materials

#### 2.1.1. Insect Samples

Samples from commercially available ready-to-eat insects were used as starting materials, to ensure that our analyses would focus on samples of actually consumed insects. Yellow mealworm larvae (*Tenebrio molitor*) and house cricket (*Acheta domesticus*) were a gift from Micronutris (http://www.micronutris.com). Silkworm pupae (*Bombyx mori*), giant worm (*Zophobas morio*) and palm worm (*Rhynchophorus ferrugineus*) larvae were purchased from Next-Food (http://www.next-food.net). Edible insect flours were prepared by carefull grinding of ready-to-eat insect samples previously frozen by soaking in liquid nitrogen.

#### 2.1.2. Chemicals

Acetonitrile Optima LC/MS grade (Fischer Scientific, Waltham, MA, USA), trifluoroacetic acid reagent plus (Sigma-Aldrich, St. Louis, MO, USA) and formic acid 98–100% LC/MS (Sigma-Aldrich), were used for the nano-LC-MS/MS analyses.

### 2.2. Edible Insect Protein Extract

Insect protein extracts were prepared by two 40 s. grinding steps of insect flour (150 mg) in Tris-HCl buffered saline, pH 7.4 (0.85 mL), in a Fast Prep-24 homogenizer (MP Biomedicals, Illkirch, France). After a centrifugation step of 15,000× *g* for 15 min. at 4 °C, the collected supernatant was filtered (0.2 µm) and stored at −20 °C until used. The bicinchoninic method kit (Pierce) [46] was used to measure the protein content in the insect protein extracts, using bovine serum albumin (Sigma) as a calibration standard.

### 2.3. Digestion and Nano-LC-MS/MS Analysis

The procedure has been described in details in [47]. After reduction and alkylation in dithiothreitol- and iodoacetamide-containing buffer, the proteome samples were loaded on 12% SDS-polyacrylamide gels. After staining with Instant Blue (Invitrogen), excised gel bands were digested in 60 µL of modified trypsin in 25 mM NH_4_HCO_3_ (10 ng/µL, Promega, sequence grade). After overnight incubation at 37 °C, the peptide mixtures were analyzed by nano-LC-MS/MS, using nanoRS UHPLC system (Dionex, Amsterdam, The Netherlands) coupled to an LTQ-Orbitrap Velos mass spectrometer (Thermo Fisher Scientific). Samples (5 mL) were loaded on a C18 precolumn (300 mm Ø × 5 mm) at 20 mL/mn in 5% acetonitrile, 0.05% TFA. After desalting, switching of the precolum to the analytical C18 colomn (75 mm Ø x 15 cm) equilibrated in 95% solvent A (5% acetonitrile, 0.2% formic acid) and 5% solvent B (80% acetonitrile, 0.2% formic acid) was performed and peptides were eluted during 105 min using a 5–50% solvent B gradient at a flow rate of 300 nL min-1. Survey scan MS spectra were acquired in the Orbitrap in the 300–2000 m/z range with resolution set to 60,000. For each survey scan, the 20 most intense ions were selected for further CID (collision-induced dissociation) fragmentation, and analysis in the linear trap. A dynamic exclusion within 60 s was used to avoid the repetitive selection of the peptides.

Database search was performed in batch mode with on all the raw files acquired for each sample, using Mascot Daemon version 2.5 (Matrix Science, London, UK). The Extract_msn.exe macro of Xcalibur (version 2.2 SP1.48, Thermo Fisher Scientific) was used to extract peak lists from Wcalibur raw files, using the following set of parameters: (1) parent ions in the 400–500 mass range, (2) no grouping of MS/MS scans, (3) threshold fixed at 1000. A peak list was generated for each analyzed gel band and Mascot search was performed for each band. Data were searched against all the entries of the Tenebrionidae 20,170,606 protein database and the genome assembly 130x_Tmol_Sep2019 (GenBank GCA_014282415.1) for *Tenebrio molitor*, the genome assembly ASM15162v1 (RefSeq GCF_000151625.1) for *Bombyx mori*, and the genome assembly LocusGenomeV1 (GenBank GCA_000516895.1) for *Locusta migratoria*. Setings for Mascot search were as follow: (1) oxidation of Met and carbamidomethylation of Cys were set as variable modifications, (2) cleavage after Lys or Arg (except before Pro) for the specificity of trypsin digestion, and a single missed trypsin cleavage was allowed, (3) mass tolerance in MS and MS/MS set to 5 ppm and O.8 Da, respectively, (4) ESI-Trap specified as intrument stetting. Mascot results were validated using the so-called “Proline” in-house software (ProFiProteomics, France) [48]. Using the target-decoy database search to control and estimate the false positive identification rate in our catalogue of insect proteins, the false discovery rate (FDR) was below 1% for peptides and proteins.

### 2.4. Bioinformatics

Multiple amino acid sequence alignments were carried out with CLUSTAL-X [49] using the stuctural Risler’s matrix for homologous residues [50].

Except for the three-dimensional structures of apolipophorin III from *Locusta migratoria* (protein data bank (PDB) code 1AEP [51] and 1LS4 [52]), the chemosensory protein from *Bombyx mori* (PDB code 2JNT) [53], the 12 kDa hemolyph protein from *Tenebrio molitor* (PDB code 1C3Z) [54] and odorant-binding proteins from Apis mellifera (PDB code 3S0D) [55] and *Bombyx mori* (PDB code 2WCJ) [56] and (PDB code 1DQE) [57], and hexamerin from *Bombyx mori* (PDB code 4L37) [58], which are available at the Protein Data Bank (PDB) [59], other apolipophorins, chemosensory proteins (CSP), odorant-binding proteins (OBP) and hexamerins, were homology modelled with YASARA structure [60], using appropriate three-dimensional structures available at the PDB as templates.

As an example, the three-dimensional structures of arylphorin from *Antheraea pernyi* (PDB code 3GWJ) [61], arylphorin from *Bombyx mori* (PDB code 4L37) [58], hemocyanin from *Panulirus japonicus* (PDB code 6L8S) [62], and hemocyanin from *Limulus polyphemus* (PDB code 1LL1) [*to be published*], were used as templates to build up to 25 different models for each of the modelled hexamerins from *Apis mellifera*, *Galleria melonella*, *Locusta migratoria* and *Tenebrio molitor*. Finally, hybrid models of hexamerins were built up from the different previous models. Similarly, the three-dimensional structures of odorant binding proteins (OBP) from *Apis mellifera* (PDB code 3S0D) [55], *Anopheles gambiae* (PDB code 3R1P) [63], AtraPBP1 from *Amyelois transtyella* (PDB code 4INW) [64], *Antheraea polyphemus* PBP1 (PDB code 2JPO) [65], *Bombyx mori* GOBP2 (PDB code 2WCJ) [56] and chemosensory protein 1 from *Bombyx mori* (PDB code 2JNT) [53]), were used as templates for the building of other OBP models from *Locusta migratoria* and *Onthophagus taurus*. Finally, hybrid models of were built up from the different previous models. Apolipophorin III from *Locusta migratoria* (code PDB 1AEP) [51], was used as a template to build the three-dimensional lodels of apolipophorin III from *Acheta domesticus*, *Bombyx mori*, *Galleria melonella*, *Schistocerca gregaria*, and *Tenebrio molitor*. Similarly, a single protein template, the crystal structure of p53 epitope-scaffold of a cysteine protease in complex with human MDM2 protein (5SWK) [66] available at the PDB, was used to build the 3D-models for the larval cuticle proteins (LCP) of *Tenebrio molitor*, *Bombyx mori*, *Locusta migratoria*, *Musca domestica*, and *Tribolium castaneum* (red flour beetle). PROCHECK [67], ANOLEA (Atomic NOn-Local Environment Assessment) [68], and the calculated QMEAN scores [69,70] were used to assess the geometric and thermodynamic qualities of the homology built three-dimensional models. Using ANOLEA, only a few residues essentially located in loops connecting the α-helices or β-sheets in the models, were found to exhibit an energy value over the fixed threshold. Similarly, the calculated QMEAN scores for all the models, gave values below 0.5.

The superposition of insect proteins was performed with Chimera [71]. Molecular cartoons were drawn with Chimera. The detection of potential cleavage sites to pepsin, trypsin and chymotrypsin, was performed at pH 1.3 for pepsin and pH 8.5 for trypsin/chymotrypsin, respectively, with the PeptideCutter web server (https://web.expasy.org/peptide_cutter/) [72], from the amino acid sequences of chemosensory proteins, odorant binding proteins, and apolipophorins III. Finally, the cleavage sites were represented on the molecular surface of the proteins, using Chimera.

## 3. Results

The nano-LC-MS/MS approach performed on insect protein extracts allowed the identification of a variable number of proteins, depending on the edible insects analyzed: 314 distinct proteins for *Bombyx mori*, 73 proteins for *Locus migratoria*, 62 proteins for *Zophobas morio*, and only 46 proteins for *Acheta domesticus*, and 42 proteins for *Rhynchophorus ferrugineus*. In a previous study, a similar approach allowed the detection of 106 distinct protein in protein extract from *Tenebrio molitor*.

As an example, (Table 1) shows the complete list of proteins identified in the silkworm (*Bombyx mori*) pupa protein extract.

Most of the potential allergens frequently distributed in edible insects, correspond in fact to IgE-binding cross-reactive allergens that occur in other groups of arthropods (acari, crustaceans), mollusks, and nematods. However, a few potential allergens including apolipophorin III, the chemosensensory protein, the coackroach allergen-like protein, hexamerin, the larval cuticle protein, the odorant binding protein and the receptor for activated protein kinase, appear as being apparently most specifically distributed in insects (Table 2).

Chemosensory proteins, odorant binding proteins and hexamerins, emerge as three groups of proteins essentially distributed in insects (≥98%), together with apolipophorins III, larval cuticle proteins and receptors for activated protein kinase, which are preferentially distributed in insects (80–85%) (Table 3). The cockroach allergen-like protein holds a unique place since its apparently occurs only in the yellow mealworm (*Tenebrio molitor*).

**Figure 1:**
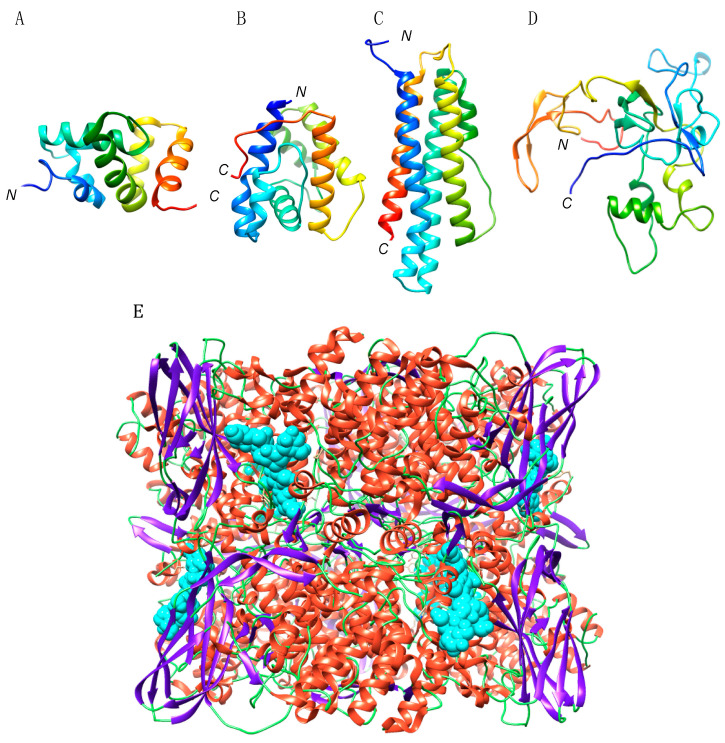
(**A**–**E**). Ribbon diagram of the three-dimensional structure/model of chemosensory protein (**A**), odorant binding protein (**B**), apolipophorin III (**C**), larval cuticle protein (**D**) and hexamerin (**E**), from *Bombyx mori*. *N* and *C* correspond to the *N*- and *C*-termini of the polypeptide chains. Fatty acids complexed to hexamerin are represented in spheres colored cyan.

Chemosensory proteins (CSP), consist of small globular proteins of 110–120 amino acids, built from 6–7 α-helices connected by short loops. They usually contain four cystein residues forming two adjacent disulfide bridge, which contribute to the tight packing of the protein (Figure 1A).

**Figure 2:**
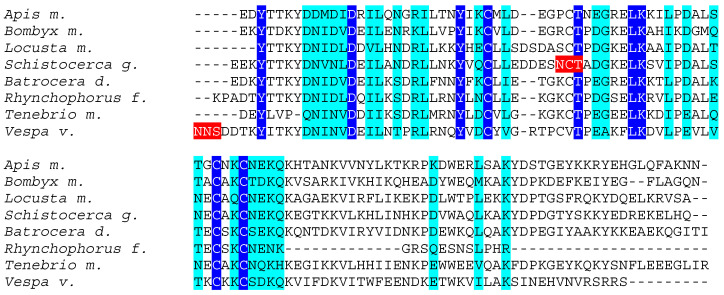
Multiple amino acid sequence alignment of chemosensory proteins from *Apis mellifera* (*Apis m.*), *Bombyx mori* (*Bombyx m.*), *Locusta migratoria* (*Locusta m.*), *Schistocerca gregaria* (*Schistocerca g.*), *Bactrocera dorsalis* (*Bactrocera d.*), *Rhynchophorus ferrugineus* (*Rhynchophorus f*.), *Tenebrio molitor* (*Tenebrio m.*), and *Vespa velutina* (*Vespa v.*). Identical amino acids are displayed in white letters highlighted in blue, homologous amino acids are displayed in black letters highlighted in cyan, and putative *N*-glycosylation sites are highlighted in red.

**Figure 3:**
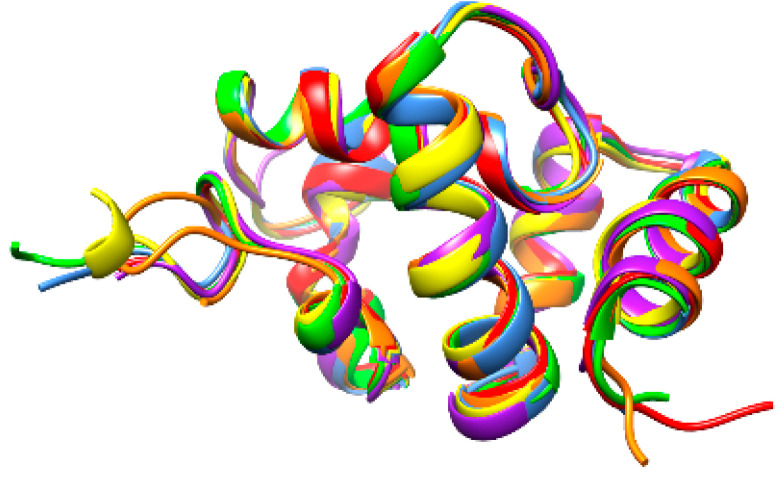
A. Superposition of the ribbon diagrams of chemosensory proteins from *Apis mellifera* (blue), *Bombyx mori* (red), *Bactrocera dorsalis* (green), *Locusta migratoria* (purple), *Schistocerca gregaria* (yellow) and *Tenebrio molitor* (orange).

Chemosensory proteins from different insect species, exhibit rather conserved amino acid sequences, especially at the *N*-terminal end of the polypeptide chain whereas their *C*-terminal end appears as less conserved (Figure 2). However, in spite of these amino acid sequence discrepancies, CSP from different insects display very similar structural organizations that are readily superposable (Figure 3).

However, in spite of these amino acid sequence discrepancies, CSP from different insects display very similar structural organizations that are readily superposable (Figure 3).

Odorant binding proteins, also known as pheromone-binding proteins, are small globular proteins with structure very similar to that of CSP, that have been recognized as potential IgE-binding proteins in yellow mealworm extracts. They typically consist of small polypeptide chains of about 120–130 amino acid residues (13–14 kDa) built up from 6 α-helices tightly packed by 3 conserved disulfide bridges (Figure 1B).

**Figure 4:**
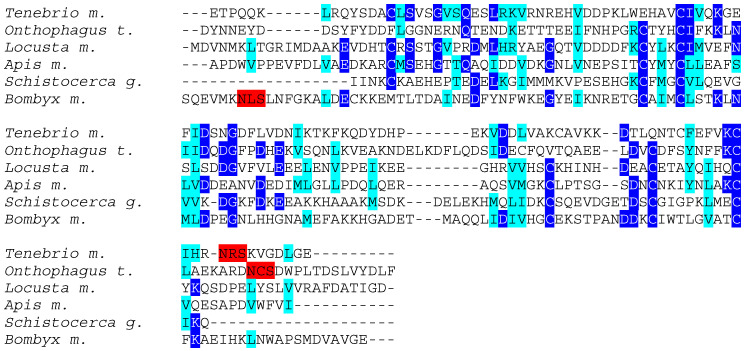
Multiple amino acid sequence alignment of odorant binding proteins (OBP) from *Tenebrio molitor* (*Tenebrio m*.), *Onthophagus taurus* (*Onthophagus t*.), Locusta migratoria (*Locusta m*.), *Apis mellifera* (*Apis m*.), *Schistocerca gregaria* (*Schistocerca g*.) and *Bombyx mori* (*Bombyx m*.). Identical amino acids are displayed in white letters highlighted in blue, homologous amino acids are displayed in black letters highlighted in cyan, and putative *N*-glycosylation sites are highlighted in red.

**Figure 5:**
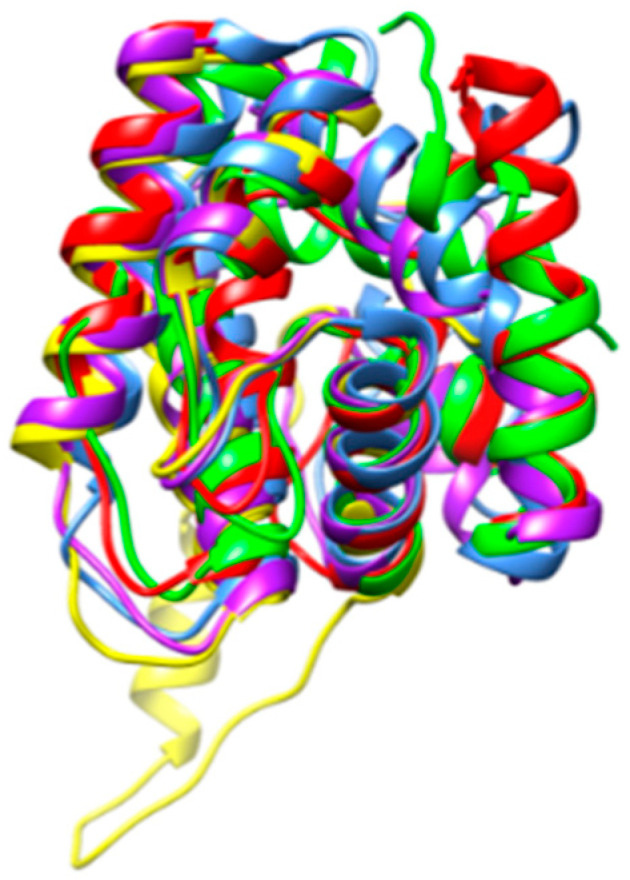
A. Superposition of the ribbon diagrams of odorant binding proteins from *Apis mellifera* (red), *Bombyx mori* (blue), *Locusta migratoria* (green), *Onthophagus taurus* (yellow) and *Tenebrio molitor* (purple).

Despite a very conserved three-dimensional structure, they differ by their amino acid sequences which show a low degree of both identity and similarity (Figure 4). Accordingly, their three-dimensional core structures are readily superposable (Figure 5).

**Figure 6:**
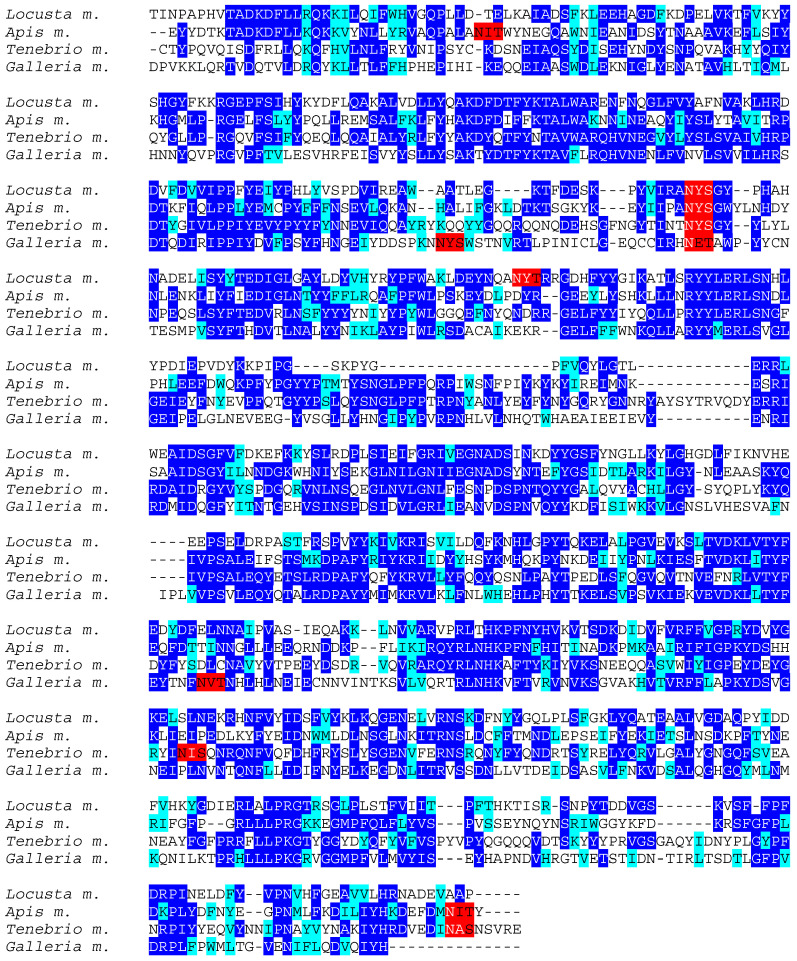
Multiple amino acid sequence alignment of hexamerins from *Locusta migratoria* (*Locusta m*.), *Apis mellifera* (*Apis m*.), *Tenebrio molitor* (*Tenebrio m*.) and *Galleria mellonella* (*Galleria m*.). Identical amino acids are displayed in white letters highlighted in blue, homologous amino acids are displayed in black letters highlighted in cyan, and putative *N*-glycosylation sites are highlighted in red.

**Figure 7:**
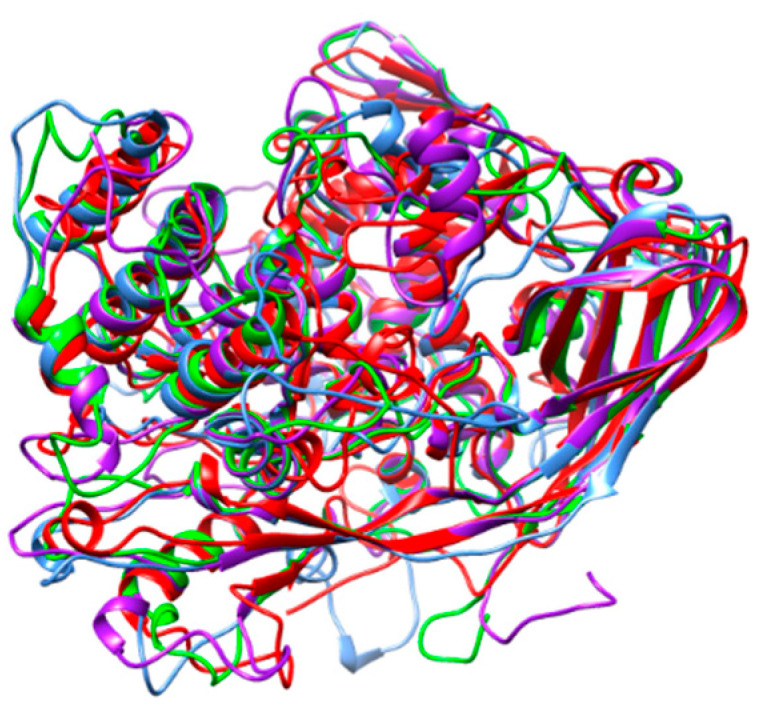
Superposition of the ribbon diagrams of hexamerin from *Apis mellifera* (red), *Galleria mellonella* (blue), *Locusta migratoria* (green), and *Tenebrio molitor* (purple).

Hexamerin consists of an insect storage protein synthesized in body fat, resulting from the non-covalent oligomerization of protomers exhibiting a hemocyanin-like domain (Figure 1E). Their amino acid sequences exhibit a high degree of identity and homology (Figure 6), which is in accordance with their very structurally conserved character. In this respect, hexamerins from different edible insects exhibit nicely superposed three-dimensional core structures, with the exception of a few exposed loops, the conformation of which differs from one structure to another (Figure 7).

**Figure 8:**
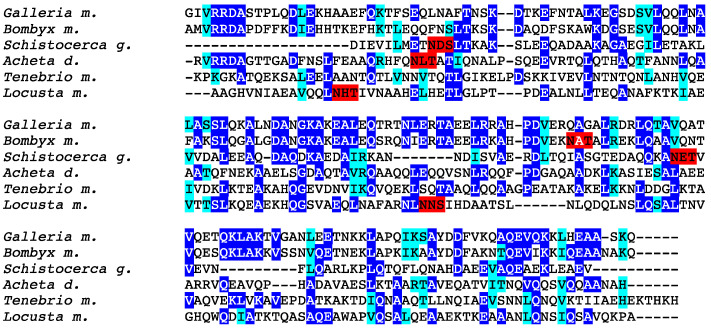
Multiple amino acid sequence alignment of apolipophorin III from *Galleria mellonella* (*Galleria m.*), *Bombyx mori* (*Bombyx m.*), *Schistocerca g.* (*Schistocerca g.*), *Acheta domesticus* (*Acheta d.*), *Tenebrio molitor* (*Tenebrio m.*) and *Locusta migratoria* (*Locusta m.*). Identical amino acids are displayed in white letters highlighted in blue, homologous amino acids are displayed in black letters highlighted in cyan, and putative *N*-glycosylation sites are highlighted in red.

**Figure 9:**
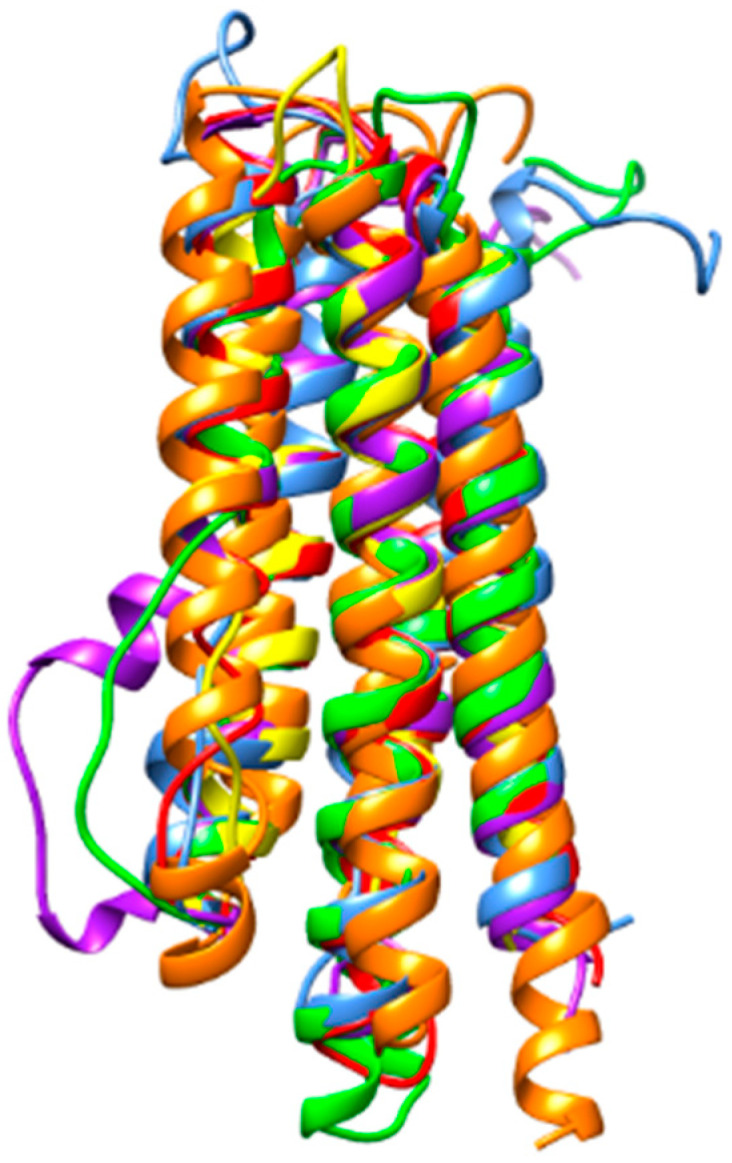
Superposition of the ribbon diagrams of apolipophorin III from *Acheta domesticus* (blue), *Bombyx mori* (green), *Locusta migratoria* (red), *Galleria mellonella* (purple), *Schistocerca gregaria* (yellow), and *Tenebrio molitor* (orange).

As previously reported, other less specific potential allergens such as apolipophorin III and larval cuticle protein, exhibit rather well conserved amino acid sequences but share a highly conserved three-dimensional structure. As an example, the multiple alignment of amino acid sequences of apolipophorin III from different edible insects shows a moderate degree of identity and similarity (Figure 8), even though their three-dimensional structures are nicely superposed (Figure 9).

In addition, it is noteworthy that different isoforms often exist for the specific allergens indentified in *Bombyx mori*, *Locusta migratoria* and *Tenebrio molitor*, as shown from the corresponding genome assemblies available for the three edible insects (Table 4). Depending on the potential allergens, the number of identified isoforms shows important variations. In this respect, proteins involved in recognition function towards environmental factors such as chemosensory proteins and odorant binding proteins, exhibit the higher diversity. This extreme diversity of both groups of chemosensory proteins and odorant binding proteins in insects, has been known for a long time.

**Figure 10:**
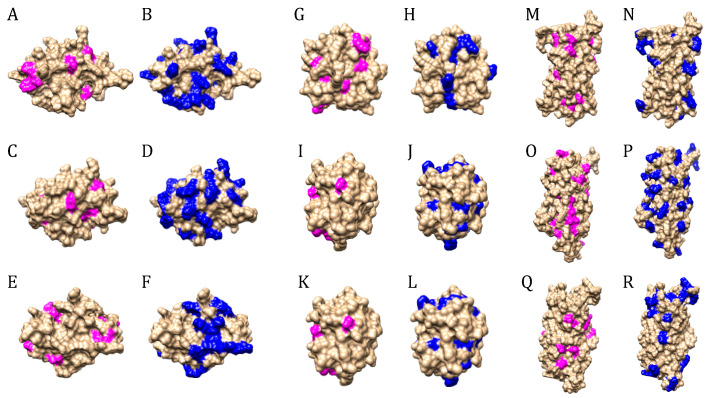
(**A–F**). Distribution of potential cleavage sites for pepsin (magenta) and trypsin (blue) on the molecular surface of chemosensory protein from *Apis mellifera* (**A,B**), *Locusta migratoria* (**C,D**) and *Tenebrio molitor* (**E,F**). (**G–L**). Distribution of potential cleavage sites for pepsin (magenta) and trypsin (blue) on the molecular surface of odorant binding protein from *Bombyx mori* (**G,H**), *Locusta migratoria* (**I,J**) and *Tenebrio molitor* (**K,L**). (**M–R**). Distribution of potential cleavage sites for pepsin (magenta) and trypsin (blue) on the molecular surface of apolipophorin III from *Acheta domesticus* (**M,N**), *Bombyx mori* (**O,P**) and *Tenebrio molitor* (**Q,R**).

Moreover, a bioinformatics identification of the potential cleavage sites for pepsin and trypsin on the molecular surface of chemosensory proteins, odorant binding proteins, and apolipophorin III, suggests the occurrence of a great number of exposed cleavage sites to trypsin and chymotrypsin, compared to the reduced number of cleavage sites accessible to pepsin (Figure 10). Accordingly, these insect protein allergens are suspected to exhibit an enhanced resistance to the proteolytic attack by pepsin and other aspartic proteases, whereas they should be further degraded at alcaline pH, in the presence of trypsin and trypsin-like proteases.

## 4. Discussion

Using an appropriate combination of SDS-PAGE and nano-LC-MS/MS proteomic analyses of proteins extracts from various insect species including the crickets *Acheta domesticus* and *Locus migratoria* (Orthoptera), the silkworm *Bombyx mori* (Hymenoptera), and the mealworms *Rhynchophorus ferrugineus*, *Tenebrio molitor* and *Zophobas morio* (Coleoptera), we have revealed the great variety of proteins occurring in insect extracts. Depending on the insect species, the number of identified proteins may vary considerably, from up to 314 distinct proteins for the *Bombyx mori* extract to only 42 proteins for the *Rhynchophorus ferrugineus* extract. These discrepancies observed in (2) the very limited availability of genome sequencing data for edible insects and, (3) possible variations in the protein content and isoform diversity among different insect species. In this respect, the more complete protein data were obtained for the well known insects *Bombyx mori* (161 distinct proteins identified), *Locusta migratoria* (73 distinct proteins identified) and *Tenebrio molitor* (106 distinct proteins identified), for which the genome sequencing data are available.

Insect allergens responsible for either contact allergies or food allergies, have been reviewed in detail by de Gier & Verhoeckx [31]. By reference to this review, it appears that most of the potential edible insect allergens consist of pan-allergens widely distributed in other arthropods, acari, chelicerates (spiders) and crustaceans, mollusks and nematods. As such, they are far from being usable as probes to detect the occurrence of insect flour in food products, due to the lack of specificity. Hopefully, a few insect allergens have been characterized as rather specific for (edible) insects since they primarily occur in insects and are much less abundant or lacking in other organisms phylogenetically-related to insects. These specific insect allergens essentially correspond to proteins dedicated to the recognition of environmental chemical signals, such as the chemosensory proteins and the odorant- or pheromone-binding proteins [73], Bla g 3 from the German cockroach (Blattella germanica) [74], and Per a 3 from the American cockroach (*Periplaneta americana*) [75], hexamerin from the edible cricket *Gryllus bimaculatus* [76], hexamerin from the maggot fly *Caliphora erythrocephala* [77], hexamerin from the fruit fly *Drosophila melanogaster* [78], and hexamerin from the yellow mealworm *Tenebrio molitor* [79]. Except for CSP and OBP, which usually are poorly glycosylated, other specific allergens of insects contain *N*-glycosylation sites and apparently consist of glycosylated proteins. Due to the different *N*-glycosylation pathways and linkage of sugar units in the insect oligosaccharides, compared to that occurring in human glycans [80,81], specific allergens from insects should act as non self CDD, responsible for some non specific immunologic-reactivity.

In spite of sharing poorly conserved amino acid sequences, all these allergens exhibit extremely well conserved three-dimensional structures. Accordingly, all these proteins are sufficiently closely-related to display an IgE-binding cross-reactivity allowing their use as specific probes for insects. Additionally, most of the identified insect specific allergens are built from a tightly packed structural fold, strengthened by disulfide bonds, that should enhance their resistance to heat denaturation susceptible to occur during the transformation processes of insect flour-containing food products. Join to their predicted resistance to acidic proteases susceptible to occur in foods and food products, all these features favor their use as particularly stable specific probes for the detection of insect flour in food products. In this respect, both processing and in vitro digestion of three mealworm species including *Tenebrio molitor*, *Zophobas atratus*, and *Alphitobius diaperinus*, and *Gryllus bimaculatus*, was reported to readily influenced their allergenic cross-reactivity [82,83,84].

With the exception of the receptor for activated protein kinase, other proteins have been previously identified as IgE-binding allergens, and could therefore be used as specific immuno-probes for the detection of added insects or insect flours to food products, in complement to other detection methods such as the genomic detection of insect proteins using polymerase chain reactions (PCR) [45] or DNA barcoding authentication [85]. Recently, functional biological bioassays based on tropomyosin as immuno-probes, have been proposed to assess the tropomyosin allergenicity of novel animal foods [86]. However, even though its allergenic character has been demonstrated [87], the cockroach-like allergen protein, which is apparently restricted to the yellow mealworm, could not serve as a relevant immuno-probe for the detection of insect proteins.

Moreover, beside the safety aspect associated to the consumption of edible insect and insect products, other important aspect dealing with the use of edible insects as food and feed, and as a protein ingredient added to improve the nutritional balance of various food products, should be considered [88,89]. Especially, the ecological and legislation aspects deserve to be discussed. Facing the increasing demand for animal proteins, edible insects and insect proteins were seen early on as a sustainable source of proteins whose production was susceptible to cause less negative impact on the environment, compared to other sources of animal proteins, e.g., the conventional forms of livestock [90,91]. Compared to the conventional sources of animal proteins, the production of insect proteins at an industrial scale, requires less energy use, releases less greenhouse gas and uses less surface area. However, while the industrial-scale production and processing of microbially- and parasitically-safe insect proteins is apparently assured, their production at a reasonable price remains a current challenge in comparison to meat or plant proteins [92,93]. In addition, outside of countries where entomophagy is traditionally developed, the reluctancy to consume edible insect often observed in other countries, especially in european countries and in USA, could be an obstacle to the development of the industrial farming of edible and insect-containg food products [94,95,96,97].

As part of the safety assessment of novel foods, two scientific opinions have been recently published by EFSA (European Food Safety Authority) on the “risk profile of insects as food and feed” [98], and on the “safety of dried yellow mealworm (*Tenebrio molitor* larva) as a novel food pursuant to Regulation (EU) 2015/2283” [99]. Both opinions, intended to help europeans politicians to put in place a regulation for the authorization of edible insects on the european market, mention the allergenic risk associated to the consumption of edible insects for allergic people. They point out the risk of reactions to either insect-specific allergens, allergens cross-reacting with other arthropods, or contaminant allergens from insect feeding such as gluten.

## 5. Conclusions

Potential IgE-binding allergens identified in edible insects, correspond essentially to pan-allergens developing some IgE-binding cross-reactivity with other homologous proteins present in other arthropods (acari, crustaceans), mollusks and nematods. Owing to this lack of specificity, they are not suitable for being used as relevant probes for the specific detection of insect flours, added as ingredients to different food products. However, a few other proteins occurring in insect protein extracts, emerge as specific allergens essentially distributed in insects whilst they are much less abundant or even lacking, in other phyllogenetically-related organisms such as acari, crustaceans, molluks and nematods.

These specific insect allergens include chemosensory proteins (CSP), odorant or pheromone- binding proteins (OBP), and hexamerin, the main storage protein of insect fat bodies. Three other proteins, apolipophorin III, the larval cuticle protein, and the receptor for activated protein kinase, could be used as specific probes since they are preferentially distributed in insects and, to a much more lesser extent, in crustaceans and nematods. The cockroach allergen-like protein is apart, because it only occurs in the yellow mealworm (*Tenebrio molitor*).

With the exception of the receptor for activated protein kinase, other proteins have been previously identified as IgE-binding allergens, and could therefore be used as specific immuno-probes for the detection of added insects or insect flours to food products, in complement to other detection methods such as the genomic detection of insect proteins using polymerase chain reactions (PCR) or the DNA barcoding authentication.

## Figures and Tables

**Table 1:** List of proteins identified in the silkworm (*Bombyx mori*) pupae protein extract. Proteins are ranked by decreasing scores. Uncharacterized proteins and fragments from identical proteins were discarded from the list.

1. Sex specific storage protein 1	55. Mitochondrial aldehyde dehydrogenase	cysteine
2. Arylphorin I	56. Hemocytin	108. Paralytic peptide binding protein
3. Sex specific storage protein 2	57. Antichymotrypsin-1	109. ATP-dependent (S)-NAD(P)H-hydrate
4. Silkworm storage protein	58. Pyruvate kinase	dehydratase
5. Arylphorine 2	59. Fructose-1,6-biphosphatase	110. Chymotrypsin inhibitor fb
6. Apolipophorin	60. 30 kDa protein	111. 30K protein 14
7. Vitellogenin	61. Chemosensory protein 7	112. Glucosamine-6-phosphate isomerase
8. Vitellogenin	62. Nucleoside diphosphate kinase	113. Thioredoxin
9. Antitrypsin isoform 1	63. Thiol peroxiredoxin	114. Adenosylhomocysteinase
10. Aliphatic nitrilase	64. Tropomyosin I	115. Proteasome subunit α type
11. Antichymotrypsin-2	65. Promoting protein	116. Carboxypeptidase
12. 30K protein 3	66. Phosphatidylethanolamine binding	117. Immune-related protein 1
13. Hemolin	protein isoform 2	118. Glutathione S-transferase δ
14. 30K protein 7	67. Obstructor-A	119. Heat shock protein 70-3
15. Low molecular 30 kDa lipoprotein	68. Mitochondrial aldehyde dehydrogenase	120. 30K protein 13
16. Hemolin	69. Actin-1	121. Heat shock cognate 70 protein
17. Major plasma protein 30K	70. Mesencephalic astrocyte-derived	122. DJ-1 β
18. Hemolin	neurotrophic factor	123. Chemosensory protein 9
19. Chitooligosaccharidolytic β-N-acetyl-	71. Malate dehydrogenase	124. Allergen
glucosaminidase	72. Transaldolase	125. 14-3-3 protein ζ
20. Odorant binding protein	73. Malic enzyme	126. 32 kDa apolipoprotein
21. Odorant-binding protein 6	74. Glyceraldehyde-3-phosphate	127. Carboxypeptidase inhibitor
22. Aliphatic nitrilase	dehydrogenase	128. Lysozyme
23. Dihydrolipoyl dehydrogenase	75. Chitinase 3	129. tRNA-nucleotidyltransferase 1
24. 30K protein 4	76. Chemosensory protein-1	130. Serpin-11
25. Carboxylic ester hydrolase	77. Serine hydroxymethyltransferase	131. Myosin heavy chain, non-muscle
26. 27 kDa glycoprotein	78. Acyl-CoA binding protein	132. Heat shock 70 kDa protein cognate 3
27. Carboxylic ester hydrolase	79. Isocitrate dehydrogenase [NADP]	133. Chemosensory protein 8
28. Tropomyosin-1 isoform 2	80. Cuticle protein	134. Fructose-1,6-biphosphatase 1
29. Low molecular mass 34 kDa lipoprotein	81. Cationic peptide CP8	135. Ubiquitin
21G1	82. Tropomyosin-1 isoforms 33/34	136. N-acetylglucosamine-6-phosphate
30. Putative peptidase	83. β-galactosidase	deacetylase
31. Saposin-like protein	84. Odorant binding protein	137. Mitochondrial cytochrome C
32. Hydroxypyruvate isomerase	85. Glyceraldehyde-3-phosphate	138. Pro-phenol oxidase
33. Serpin-2	dehydrogenase	139. Catalase
34. Serpin-6	86. Tenebrin	140. Glutathione-S-Transferase 1
35. Glucosamine-6-phosphate isomerase	87. Aminoacylase	141. Thioredoxin peroxidase
36. Serpin-9	88. ARP-like protein	142. Peroxiredoxin 1
37. Serpin-2	89. Serpin-7	143. Glutamate dehydrogenase
38. Hemolymph juvenile hormone binding	90. Serpin-3	144. Nucleoplasmin-like protein
protein	91. Calmodulin	145. Angiotensin converting enzyme
39. Molting fluid carboxypeptidase A	92. Imaginal disk growth factor	146. DNA supercoiling factor
40. Superoxide dismutase [Cu-Zn]	93. Actin-depolymerizing factor 1	147. Nucleoside diphosphate kinase
41. Fructose-biphosphate aldolase	94. Chymotrypsin inhibitor SCI-III	148. HSP70
42. Fibrillin-like protein	95. Myosin light chain 2	149. Sericin 2
43. Bm8 interacting protein 2d-4	96. POX-C	150. Molting carboxypeptidase A
44. Superoxide dismutase [Cu-Zn]	97. Scarface	151. Fibrillin-1
45. Type IV collagen	98. Proteasome subunit α type	152. Small heat shock protein 20.8
46. Glyceraldehyde-3-phosphate	99. Chymotrypsin inhibitor SCI-I	153. β-glucuronidase
dehydrogenase	100. Kazal-type proteinase inhibitor	154. Trehalase
47. Serpin-5	101. Polyubiquitin-c isoform x7	155. DNA (apurinic or apyrimidinic site)
48. Superoxide dismutase [Cu-Zn]	102. Chymotrypsin inhibitor SCI-II	lyase
49. Actin	103. Carboxylic ester hydrolase	156. Cuticlin-1
50. Thioredoxin	104. Prophenoloxidase subunit 2	157. Kv-channel-interacting protein
51. Glucosamine-6-phosphate isomerase	105. Thiol peroxiredoxin	158. Ferritin
52. Cystathionine γ-lyase	106. Ubiquitin/ribosomal protein S27 Ae	159. Chemosensory protein 5
53. Retinoic acid binding protein	fusion protein	160. Arylphorin subunit α
54. Putative actin-related protein	107. Secreted protein acidic and rich in	161. Pterin carbinolamin dehydratase

**Table 2:** List of IgE-binding cross-reactive allergens identified (+) in edible insects (Bm: *Bombyx mori*, Tm: *Tenebrio molitor*, Ad: *Acheta domesticus*, LM: *Locusta migratoria*, Zm: *Zophobas morio*, Rf: *Rhynchophorus ferrugineus*) shared (X) with crustaceans (C), acari (Ac), insects (I), mollusks (M), worms (W), fungi (F), plants (P) and animals (A). The allergens more specifically distributed in insects are black boxed **X**.

Allergen	Bm	Tm	Ad	Lm	Zm	Rf	C	Ac	I	M	W	F	P	An
Acidic ribosomal protein				+	+	+	X	X	X	X	X	X	X	X
Actin α	+	+	+	+	+	+	X	X	X	X	X	X	X	X
Actinin		+	+				X	X	X	X	X	X	X	X
Adenosylhomocysteinase	+								X				X	X
α-Amylase		+					X	X	X	X	X	X	X	X
Apolipophorin III	+	+	+						**X**	X	X	X	X	X
Apolipoprotein	+						X	X	X	X	X	X	X	X
Arginine kinase	+	+	+	+	+	+	X	X	X	X	X			X
Arylphorin, Hemocyanin	+	+		+	+		X		**X**					
Aspartic protease		+					X	X	X			X	X	X
ATP synthase			+	+	+		X	X	X	X	X	X	X	X
Carboxypeptidase	+						X	X	X	X	X	X	X	X
Catalase	+						X	X	X	X	X	X	X	X
Chemosensory protein	+			+		+	X	X	**X**				X	X
Chitinase	+	+					X	X	X	X	X	X	X	X
Cockroach allergen-like protein		+							**X**					
Cystatin proteinase inhibitor	+						X	X	X	X	X	X	X	X
Cytochrome C	+				+	+	X	X	X	X	X	X	X	X
Enolase	+	+	+	+	+		X	X	X	X	X	X	X	X
Fatty acid-binding protein	+	+		+		+	X	X	X	X	X	X	X	X
Ferritin	+						X	X	X	X	X	X	X	X
Fructose-1,6-biphosphate aldolase	+	+	+	+	+	+			X	X		X		
Glucosamine-6-phosphate isomerase	+						X	X	X	X	X	X	X	X
Glutathione S-transferase	+	+					X	X	X	X	X	X	X	X
Glyceraldehyde-3-phosphate dehydrogenase	+	+	+	+	+	+	X	X	X	X	X	X	X	X
Hexamerin	+	+		+	+	+	X		**X**				X	
HSP 70	+	+	+	+	+		X	X	X	X	X	X	X	X
Larval cuticle protein	+	+				+	X	X	**X**		X			X
Lipocalin		+					X	X	X	X	X	X	X	X
Lysosomal aspartic protein		+					X	X	X	X	X	X	X	X
Lysozyme	+						X	X	X	X	X	X	X	X
Malate dehydrogenase	+	+	+				X	X	X	X	X	X	X	X
Mitochondrial aldehyde dehydrogenase	+						X	X	X	X	X	X	X	X
α-Myosin		+	+	+	+	+			X					X
Myosin heavy chain	+	+					X	X	X	X	X	X	X	X
Myosin light chain	+	+					X	X	X	X	X	X	X	X
Nucleoside diphosphate kinase	+						X	X	X	X	X	X	X	X
Odorant-binding protein	+	+	+	+	+	+	X	X	**X**		X		X	X
Paramyosin long form		+	+				X		X		X	X	X	X
Paramyosin short form		+	+				X	X	X		X	X	X	X
Peroxiredoxin	+	+					X	X	X	X	X	X	X	X
Pyruvate kinase	+	+	+				X	X	X	X	X	X	X	X
Receptor for activated protein kinase		+					X	X	**X**	X	X	X	X	X
Sarcoplasmic calcium-binding protein		+					X	X	X	X		X	X	X
Serine protease		+					X	X	X	X	X	X	X	X
Serpin	+	+					X	X	X	X	X	X	X	X
Superoxide dismutase [Cu-Zn]	+	+	+				X	X	X	X	X	X	X	X
Thioredoxin	+	+					X	X	X	X	X	X	X	X
Transaldolase	+						X	X	X	X	X	X	X	X
Triosephosphate isomerase		+			+		X	X	X	X	X	X	X	X
Tropomyosin 1	+	+	+	+	+	+	X	X	X	X	X	X	X	X
Tropomyosin 2		+	+	+			X	X	X	X	X	X	X	X
Troponin C		+			+		X	X	X	X	X	X	X	X
Troponin T		+	+		+		X	X	X	X	X	X	X	X
Trypsin		+					X	X	X	X	X	X	X	X
Tubulin α		+	+				X	X	X	X	X	X	X	X
Tubulin β		+	+	+	+		X	X	X	X	X	X	X	X
Vitellogenin	+	+		+			X	X	X	X	X	X	X	X

**Table 3:** Number and proportion (expressed as %) of specific proteins identified in insects, crustaceans, mollusks and nematods. N/I: protein not identified. Green: highly specific insect proteins.

Protein:	Insects:	Crustaceans:	Mollusks:	Nematods:
Apolipophorin III	549 (79.7%)	6 (0.8%)	26 (3.8%)	108 (15.7%)
Chemosensory protein	17,207 (98%)	142 (0.8%)	47 (0.2%)	180 (1%)
Cockroach allergen-like protein	1	N/I	N/I	N/I
Hexamerin	395 (99.5%)	2 (0.5%)	N/I	N/I
Larval culticle protein	3971 (84.7%)	315 (6.7%)	1 (0.02%)	399 (8.5%)
Odorant binding protein	14,318 (99.8%)	13 (0.08%)	N/I	19 (0.13%)
Receptor for activated protein kinase	313 (79%)	16 (4%)	17 (4.4%)	50 (12.6%)

**Table 4:** Number of specific protein isoforms identified in *Bombyx mor*i, *Locusta migratoria*, and *Tenebrio molitor*, from the genome assemblies available at the NCBI database.

Protein:	*Bombyx mori*	*Locusta migratoria*	*Tenebrio molitor*
Chemosensory protein	16	28	12
Odorant binding protein	23	26	19
Hexamerin	0	7	2
Apolipophorin III	3	6	1
Larval culticle protein	9	3	10
